# How to Sit

**Published:** 1878-06

**Authors:** 


### HOW TO SIT.

ALL consumptive people, and all afflicted with spinal deformities, sit habitually crooked, in one or more curves of the body. There was a time in all these when the body had its natural erectness, when there was the first departure on the road to death. The make of our chairs, especially that great barbarism, the unwieldy and disease-engendering rocking chair, favors these diseases, and undoubtedly, in some instances, leads to bodily habits which originate the ailments just named, to say nothing of piles, fistula, and the like. The painful or sore feeling which many are troubled with incessantly for years, at the extremity of the backbone, is the result of sitting in such a position that it rests upon the seat of the chair, at a point several inches forward of the chair back. A physiological chair, one which shall promote the health and preserve the human form erect and manly as our Maker made it, should have the back straight, at right-angles with the seat, the seat itself not being over eight inches deep. A chair of this kind will do more towards correcting the lounging habits of our youth than multitudes of parental lecturings, for then if they are seated at all they must sit erect, otherwise there is no seat-hold.

In partial connection with this subject, Dr. Potter said, in a recent address at Albany, on

